# Prevalence, Trends, and Associated Factors of HIV, Syphilis, and Anti–Hepatitis C Virus Seropositivity Among Attendees at Voluntary Counseling and Testing Clinics in Zhejiang Province, China, From 2022 to 2024: Retrospective Quantitative Study

**DOI:** 10.2196/96584

**Published:** 2026-09-29

**Authors:** Jinlei Zheng, Lin Chen, Lin He, Tingting Jiang, Hui Wang, Wanjun Chen, Yapin Yao

**Affiliations:** 1 Department of HIV/AIDS and STDs Control and Prevention Zhejiang Provincial Center for Disease Control and Prevention Hangzhou, Zhejiang China

**Keywords:** human immunodeficiency virus, HIV, hepatitis C virus, HCV, syphilis, human immunodeficiency virus–syphilis coinfection, HIV-syphilis coinfection, human immunodeficiency virus–hepatitis C virus coinfection, HIV-HCV coinfection

## Abstract

**Background:**

In the postpandemic era, trends in HIV, syphilis, and hepatitis C virus (HCV) infections, as well as the prevalence of coinfections among high-risk groups, may have shifted; however, relevant empirical studies are limited.

**Objective:**

This study aimed to assess the seropositivity rates and coinfection rates for HIV, syphilis, and anti-HCV; and to explore the correlates of HIV, syphilis, and anti-HCV positivity among voluntary counseling and testing (VCT) clients at Centers for Disease Control and Prevention (CDC)–affiliated clinics in Zhejiang Province from 2022 to 2024.

**Methods:**

This retrospective study analyzed routine data extracted in January 2025 from the National HIV Information Prevention and Control System. The Cochran-Armitage trend chi-square test was used to analyze trends in positivity rates over the years. Logistic regression was used to identify relevant indicators for positivity for HIV, syphilis, and anti-HCV in 2024.

**Results:**

The HIV positivity rates decreased from 2.462% (743/30,182) in 2022 to 1.664% (681/40,916) in 2024 (*χ*^2^_1_ for trend =53.6; *P*<.001). The syphilis positivity rates were 1.378% (414/30,049), 1.502% (675/44,951), and 1.62% (647/39,929) in 2022, 2023, and 2024, respectively (*χ*^2^_1_ for trend=6.8; *P*=.03). The anti-HCV positivity rates were 0.309% (77/24,950), 0.387% (161/41,593), and 0.504% (192/38,098) in 2022, 2023, and 2024, respectively (*χ*^2^_1_ for trend=14.8; *P*<.001). In 2024, the positivity rates for dual HIV–anti-HCV coinfection, dual HIV-syphilis coinfection, and triple HIV-syphilis–anti-HCV coinfection were 0.011%, 0.203%, and 0.005%, respectively. Multivariable logistic regression analysis showed that age ≥35 years and HIV testing history were associated with HIV, syphilis, and anti-HCV positivity; compared with proactive consultation, outreach consultation was associated with lower odds of HIV positivity (adjusted odds ratio [aOR] 0.096, 95% CI 0.060-0.153), but higher odds of syphilis positivity (aOR 1.241, 95% CI 1.033-1.491) and anti-HCV positivity (aOR 2.837, 95% CI 1.882-4.276); compared with consulting for casual heterosexual behavior, consulting for homosexual behavior was associated with higher odds of HIV positivity (aOR 1.909, 95% CI 1.514-2.408) and lower odds of anti-HCV positivity (aOR 0.031, 95% CI 0.011-0.082).

**Conclusions:**

From 2022 to 2024 in Zhejiang Province, the HIV and anti-HCV positivity rates among VCT clients at CDC-affiliated clinics declined, while the syphilis positivity rates increased. The coinfection positivity rates for these 3 infections remained at low levels. Individuals reporting homosexual behavior constituted a high-risk group for HIV positivity yet exhibited a lower positivity rate for anti-HCV. Among CDC-affiliated VCT clients, older age, lower educational attainment, and prior HIV testing history were associated with higher infection positivity rates, suggesting that these characteristics might help refine targeting of VCT and prevention efforts within this setting.

## Introduction

To further strengthen the prevention and control of HIV-AIDS, the Joint United Nations Programme on HIV-AIDS set an ambitious 95-95-95 target for 2025: specifically, 95% of people within the subpopulation who are living with HIV know their HIV status, 95% of people living with HIV in key populations who know their HIV status are on treatment, and 95% of people within the subpopulations who are on treatment have suppressed viral loads [1]. Over the past decade, the treatment rates and viral suppression rates among people living with HIV in China have risen rapidly. Antiretroviral therapy (ART) coverage among diagnosed people living with HIV has approached 90%, whereas more than 94% of people living with HIV receiving treatment for at least 12 months have achieved viral suppression. However, the “detection rate of diagnosed cases” has remained <90% [2]. Consequently, HIV testing strategies have been continuously refined, with voluntary counseling and testing (VCT) serving as a crucial component.

HIV VCT refers to a process in which individuals who suspect they are at risk of HIV infection consult professionals and voluntarily undergo HIV testing along with related referrals and extended services with full informed consent and complete confidentiality [3]. Zhejiang Province has provided HIV VCT services since 2003 through facilities, including Centers for Disease Control and Prevention (CDC), general hospitals, and maternal and child health hospitals. As of 2024, there were 351 HIV VCT clinics in Zhejiang: 94 located in CDC facilities, 143 in general hospitals, 78 in maternal and child health hospitals, and 36 in community health service centers and township health centers, yielding a cumulative testing volume of approximately 90,000 person-times annually. In 2017, hepatitis C virus (HCV) antibody screening was incorporated into HIV VCT clinics, and in 2022, HCV testing was officially integrated into the HIV Testing and Counseling Information Management System.

HIV VCT plays a pivotal role in HIV-AIDS prevention and control [4-7]. According to an analysis by Chen et al [4], attendees of VCT and sexually transmitted disease clinics accounted for merely 4.5% of all individuals undergoing HIV testing, but they represented 23.7% of all HIV-positive cases identified in 2018. A study conducted in Turkey using an HIV transmission model projected that a 2-fold and 4-fold increase in the number of both VCT and other testing facilities would reduce the annual incidence by 71% and 91%, respectively, by 2033, whereas HIV prevalence was estimated to be 34% and 51% lower, respectively, than that in the scenario of taking no action [5].

With the release of guidelines by the World Health Organization and the rollout of national HCV elimination plans in China to eliminate the public health threat of HCV, coinfection with HIV, syphilis, and HCV has become a research focus [8-10]. Multiple studies have shown that the prognosis of patients with HIV coinfected with other diseases is poorer than that of patients with HIV monoinfection [11-14]. For instance, a study by Fan et al [12] demonstrated that patients with HIV-syphilis coinfection exhibited smaller increases in CD4+ T-cell counts and cluster of differentiation 4 (CD4)–cluster of differentiation 8 (CD8) ratios than patients with HIV monoinfection. HIV-HCV coinfection not only accelerates the progression of AIDS and increases the risk of liver-related end points but also affects the selection of ART regimens for AIDS, thereby compromising patient survival and prognosis [13,14]. Therefore, with the advancement of HIV-AIDS prevention and control efforts, the innovative strategy of “integrated prevention and control of multiple diseases” has been proposed globally [15].

To better characterize the prevalence of HIV, syphilis, and HCV monoinfection and coinfection among high-risk populations in Zhejiang Province, this study performed an in-depth analysis using data collected between 2022 and 2024 at CDC-affiliated VCT clinics. The dataset was large-scale and highly representative. These clinics were selected primarily because the data they generate are of higher quality than those obtained from other health care facilities. In addition, VCT clients at CDC-affiliated clinics are high-risk individuals who seek services proactively or are reached through outreach programs; these individuals exhibit multiple high-risk behaviors, making CDC-affiliated VCT clinics a critical data source for investigating the positivity rates of HIV, syphilis, and anti-HCV.

## Methods

### Study Population

Individuals who received HIV counseling and testing at CDC-affiliated VCT clinics in Zhejiang Province from 2022 to 2024 were included in this analysis. Individuals who received only counseling but did not undergo HIV testing were excluded. A total of 30,182, 46,060, and 40,916 individuals were enrolled in 2022, 2023, and 2024, respectively. Due to inconsistent implementation of multipathogen testing across VCT clinics, some VCT clinics did not offer syphilis or anti-HCV testing services during these 3 years: HIV test volumes were 30,182, 46,060, and 40,916; syphilis test volumes were 30,049, 44,951, and 39,929; and anti-HCV test volumes were 24,950, 41,593, and 38,098 in 2022, 2023, and 2024, respectively. The numbers of participants tested for HIV, syphilis, and anti-HCV during the same visit in 2022, 2023, and 2024 were 24,909, 41,563, and 38,065, respectively, accounting for 82.5%, 90.2%, and 93% of all VCT clients in each year.

### Study Design and Data Collection

This was a retrospective analysis of repeated annual cross-sectional data from routine VCT surveillance. Data were obtained from the National HIV/AIDS Information Prevention and Control System. The VCT Clinics Database of Zhejiang Province was downloaded in January 2025 and screened using the following criteria: (1) data submission years covered 2022 to 2024; (2) the reporting institution was a CDC facility; and (3) complete HIV test results were available. It was not until 2022 that syphilis and anti-HCV test results were added to VCT data collection nationwide. The dataset included submissions from 94 CDC sites in Zhejiang Province. Data were submitted by all 94 CDC-affiliated VCT clinics across the province, achieving full data coverage throughout the study period.

### Measures

In this study, HIV positivity was defined as testing positive on a confirmed HIV test (Western blot confirmation); syphilis positivity was defined as testing positive for both specific and nonspecific antibodies (rapid plasma regain [RPR]–toluidine red unheated serum test [TRUST] and Treponema pallidum particle agglutination assay [TPPA]–enzyme-linked immunosorbent assay [ELISA]); and anti-HCV positivity was defined as testing positive for HCV antibodies (ELISA).

Relevant information on counseling region, gender, age, marital status, and educational background was analyzed to assess the sociodemographic characteristics of participants. The 11 counseling regions were divided into 3 groups based on the level of economic development: high-income regions included Hangzhou and Ningbo; middle-income regions included Wenzhou, Jiaxing, Shaoxing, and Taizhou; and low-income regions included Quzhou, Lishui, Zhoushan, and Huzhou.

Consultation types were divided into 3 categories: proactive, outreach, and referral consultation. Proactive consultation covers clients who voluntarily present at CDC-affiliated VCT clinics for HIV counseling and testing. Outreach consultation includes individuals who received counseling and testing during CDC on-site outreach activities. Referral consultation refers to clients who attend CDC-affiliated VCT clinics for HIV counseling and testing after being referred by other agencies or peers, such as medical institutions, family planning institutions, maternal and child health institutions, or community-based organizations (CBOs).

The primary reasons for consultation included a history of casual heterosexual behavior, homosexual behavior, blood exposure–related behaviors, having an HIV-positive intimate partner (spouse or regular partner), and injection drug use (IDU). Histories of plasma donation, surgery, and blood transfusion, or blood product use were grouped under blood exposure–related behaviors. The option “others” included a history of occupational exposure, maternal HIV positivity, no history of high-risk behavior, and other reasons not mentioned.

### Statistical Analysis

Data were analyzed using SPSS (version 25.0; IBM Corp). Data from 2022 to 2024 were used to analyze the demographic characteristics and the positivity rates for HIV, syphilis, and anti-HCV; data from 2024 were used to analyze the predictors associated with these positivity rates. The calculation of positivity rates and influencing factors was based on the aforementioned data.

Descriptive analyses were used to describe the demographic characteristics of VCT clients at CDC-affiliated clinics, and statistical differences between groups were analyzed using the chi-square test. The Cochran-Armitage trend test or the chi-square test for trend was used for comparing proportions across years. Bar and line charts were used to display the test volumes, number of positive cases, and positivity rates for HIV, syphilis, and anti-HCV.

Missing values in marital status and education (approximately one‑third of observations) were addressed using multiple imputation under the missing‑at‑random (MAR) assumption. Twenty imputed datasets were generated, incorporating the binary outcome variable and all covariates included in subsequent regression models. Univariable and multivariable binary logistic regression (LR) models (backward LR) were fitted separately on each imputed dataset. Pooled odds ratios (OR), adjusted odds ratios (aORs), 95% CIs, and *P* values were combined across imputed datasets using the Rubin rules, accounting for both within‑ and between‑imputation variance. Complete‑case analysis and an additional sensitivity analysis treating “unknown” as an independent category were performed to evaluate the robustness of the regression findings. *P*<.05 was considered to indicate statistical significance.

### Ethical Considerations

The study was conducted in accordance with the Declaration of Helsinki, and all procedures were approved by the ethics committee of the Zhejiang Provincial Center for Disease Control and Prevention (2026-038-01). Ethics approval for the waiver of informed consent was granted because the data were routinely collected by province-wide VCT clinics for daily clinical service delivery, rather than collected specifically for a research project.

## Results

A total of 30,182, 46,060, and 40,916 VCT clients were enrolled at CDC-affiliated clinics in Zhejiang Province in 2022, 2023, and 2024, respectively. In 2024, the proportions of individuals in high-income, middle-income, and low-income regions were 27.7% (n=11,335), 53% (n=21,671), and 19.3% (n=7910), respectively. Of these clients, 78.8% (n=32,228) were male, 52.1% (n=21,305) were aged 35 years, and 39.8% (n=16,267) were married. In total, 19.6% (n=8000) had a college education or higher. Of all respondents, 74.1% (n=30,307) received proactive consultation, 21.7% (n=8893) received outreach consultation, and 4.2% (n=1716) received referral consultation. For consultation reasons, 43% (n=17,601) consulted for homosexual behavior, followed by casual heterosexual behavior (n=11,972, 29.3%) and HIV-positive intimate partners (n=2077, 5.1%). Differences in these characteristics among the 3 years were statistically significant (*P*<.05; Table 1).

As shown in Figures 1 to 3, an analysis of the positivity rates for HIV, syphilis, and anti-HCV from 2022 to 2024 revealed the following: the HIV positivity rates decreased from 2.462% (743/30,182) in 2022 to 1.664% (681/40,916) in 2024, with a significant difference according to the Cochran-Armitage trend test (*χ*^2^_1_ for trend=53.6; *P*<.001; Figure 1). Between 2022 and 2024, the syphilis positivity rates were 1.378% (414/30,049), 1.502% (675/44,951), and 1.62% (647/39,929), respectively, with a significant difference according to the chi-square test for trend (*χ*^2^_1_ for trend=6.8; *P*=.03; Figure 2). The anti-HCV positivity rates were 0.309% (77/24,950), 0.387% (161/41,593), and 0.504% (192/38,098) in 2022, 2023, and 2024, respectively, with a significant difference according to the chi-square test for trend (*χ*^2^_1_ for trend=14.8; *P*<.001; Figure 3).

From 2022 to 2024, the positivity rates for dual HIV-syphilis coinfection were 0.3% (90/30,049), 0.249% (112/44,951), and 0.203% (93/39,929), respectively; those for dual HIV–anti-HCV coinfection were 0.012% (3/24,950), 0.002% (1/41,593), and 0.011% (4/38,098), respectively; and the positivity rates for triple HIV-syphilis–anti-HCV coinfection were 0.008% (2/24,909), 0% (0/41,563), and 0.005% (2/38,065), respectively (Table 2).

The results of univariable and multivariable analyses of factors associated with HIV, syphilis, and anti-HCV positivity are shown in Table 3.

Compared with high-income regions, middle-income regions had higher odds of HIV positivity (aOR 1.388, 95% CI 1.161-1.660) and anti-HCV positivity (aOR 3.568, 95% CI 1.924-6.615), while low-income regions had lower odds of HIV positivity (aOR 0.411, 95% CI 0.301-0.561). Female participants had a lower likelihood of HIV infection (aOR 0.511, 95% CI 0.385-0.676). The odds of HIV, syphilis, and anti-HCV positivity all rose with advancing age. Compared with unmarried clients, being married was associated with lower odds of HIV positivity (aOR 0.565, 95% CI 0.432-0.738) and higher odds of anti-HCV positivity (aOR 5.109, 95% CI 2.904-8.990).

Compared with participants with junior high school education or below, those with senior high school (aOR 0.455, 95% CI 0.356-0.583) or college education or above (aOR 0.646, 95% CI 0.515-0.811) had lower odds of HIV positivity; those with senior high school (aOR=0.665, 95% CI 0.541-0.818) or college education or above (aOR=0.734, 95% CI 0.584-0.922) had lower odds of syphilis positivity; and those with senior high school (aOR=0.448, 95% CI 0.254-0.792) or college education or above (aOR=0.392, 95% CI 0.228-0.672) had lower odds of anti-HCV positivity. Compared with proactive consultation, outreach consultation was associated with lower odds of HIV positivity (aOR 0.096, 95% CI 0.060-0.153), but higher odds of syphilis positivity (aOR 1.241, 95% CI 1.033-1.491) and anti-HCV positivity (aOR 2.837, 95% CI 1.882-4.276). Referral consultation was associated with higher odds of HIV positivity (aOR 1.632, 95% CI 1.249-2.131) and anti-HCV positivity (aOR 31.869, 95% CI 20.557-49.406).

Compared with participants seeking consultation for casual heterosexual behavior, those consulting for homosexual behavior had higher odds of HIV positivity (aOR 1.909, 95% CI 1.514-2.408) and lower odds of anti-HCV positivity (aOR 0.031, 95% CI 0.01-0.082). Participants consulting as intimate partners of HIV-positive individuals had higher odds of HIV positivity (aOR 3.526, 95% CI 2.546-4.882). Those consulting for IDU had higher odds of anti-HCV positivity (aOR 30.495, 95% CI 15.584-59.672).

Participants who had never been tested for HIV had higher odds of HIV positivity (aOR 1.455, 95% CI 1.208-1.754), syphilis positivity (aOR 1.294, 95% CI 1.105-1.516), and anti-HCV positivity (aOR 2.943, 95% CI 2.069-4.187).

**Table 1:** Sociodemographic characteristics of voluntary counseling and testing clients at Centers for Disease Control and Prevention–affiliated clinics from 2022 to 2024.

Variables			2022 (n=30,182), n (%)		2023 (n=46,060), n (%)		2024 (n=40,916), n (%)		*χ*^2^ (df)			*P* value	
**Regions**										201.7 (4)			<.001
	High-income	9201 (30.5)		14,314 (31.1)		11,335 (27.7)							
	Middle-income	14,769 (48.9)		23,364 (50.7)		21,671 (53)							
	Low-income	6212 (20.6)		8382 (18.2)		7910 (19.3)							
**Sex**										328.9 (2)			<.001
	Male	22,053 (73.1)		34,631 (75.2)		32,228 (78.8)							
	Female	8129 (26.9)		11,429 (24.8)		8688 (21.2)							
**Age (years)**										252.1 (8)			<.001
	16-24	5321 (17.6)		7788 (16.9)		6843 (16.7)							
	25-34	10,273 (34)		15,914 (34.6)		12,768 (31.2)							
	35-44	7061 (23.4)		10,506 (22.8)		9583 (23.4)							
	45-54	4532 (15)		6638 (14.4)		6472 (15.8)							
	≥55	2995 (9.9)		5214 (11.3)		5250 (12.8)							
**Marital status**										345.0 (6)			<.001
	Unmarried	9196 (30.5)		13,635 (29.6)		12,501 (30.6)							
	Married	12,304 (40.8)		16,716 (36.3)		16,267 (39.8)							
	Divorced or widowed	1232 (4.1)		2016 (4.4)		1683 (4.1)							
	Unknown	7450 (24.7)		13,693 (29.7)		10,465 (25.6)							
**Educational background**										334.5 (6)			<.001
	Junior high school and under	10,301 (34.1)		13,487 (29.3)		12,910 (31.6)							
	Senior high school	6466 (21.4)		9196 (20)		8367 (20.4)							
	College and above	5320 (17.6)		8961 (19.4)		8000 (19.6)							
	Unknown	8095 (26.8)		14,416 (31.3)		11,639 (28.4)							
**Consultation types**										775.6 (4)			<.001
	Proactive	22,993 (76.2)		31,233 (67.8)		30,307 (74.1)							
	Outreach	5882 (19.5)		12,531 (27.2)		8893 (21.7)							
	Referral	1307 (4.3)		2296 (5)		1716 (4.2)							
**Consultation reasons**										2047.4 (10)			<.001
	Casual heterosexual behavior	12,575 (41.7)		16,237 (35.2)		11,972 (29.2)							
	Homosexual behavior	10,024 (33.3)		17,447 (37.9)		17,601 (43)							
	Blood exposure–related behaviors	267 (0.9)		859 (1.9)		241 (0.6)							
	Having an HIV-positive intimate partner	2008 (6.7)		2063 (4.5)		2077 (5.1)							
	Injection drug use	194 (0.6)		307 (0.7)		74 (0.2)							
	Others	5114 (16.9)		9147 (19.8)		8951 (21.9)							
**HIV testing history**										211.1 (2)			<.001
	No	17,149 (56.8)		26,452 (57.4)		21,601 (52.8)							
	Yes	13,033 (43.2)		19,608 (42.6)		19,315 (47.2)							

**Figure 1:**
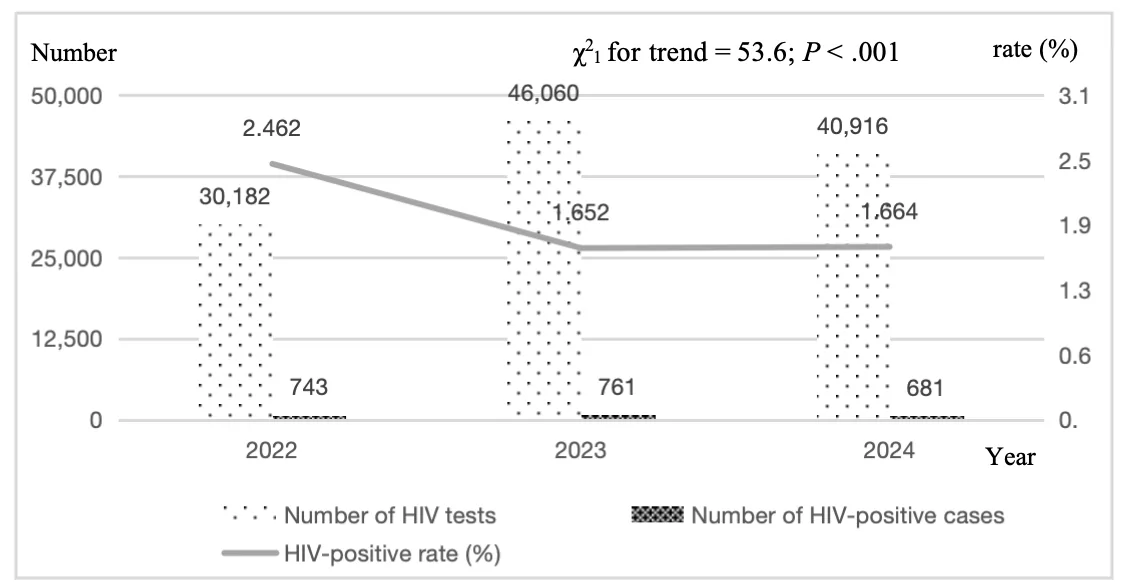
Trends in HIV test volume, HIV-positive cases, and HIV positivity rate among voluntary counseling and testing clients at Centers for Disease Control and Prevention–affiliated clinics in Zhejiang Province from 2022 to 2024, together with the results of the chi-square test for trend analysis.

**Figure 2:**
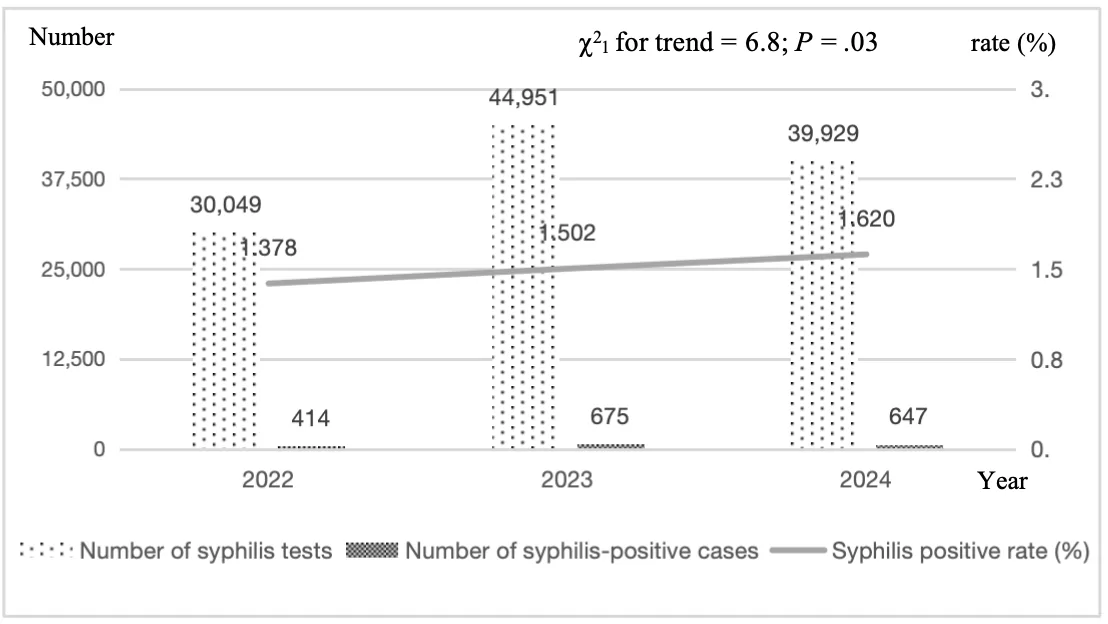
Trends in syphilis testing volume, syphilis-positive cases, and syphilis positivity rate among voluntary counseling and testing clients at Centers for Disease Control and Prevention–affiliated clinics in Zhejiang Province from 2022 to 2024. The statistical significance of the temporal trends was examined using the chi-square test for trend.

**Figure 3:**
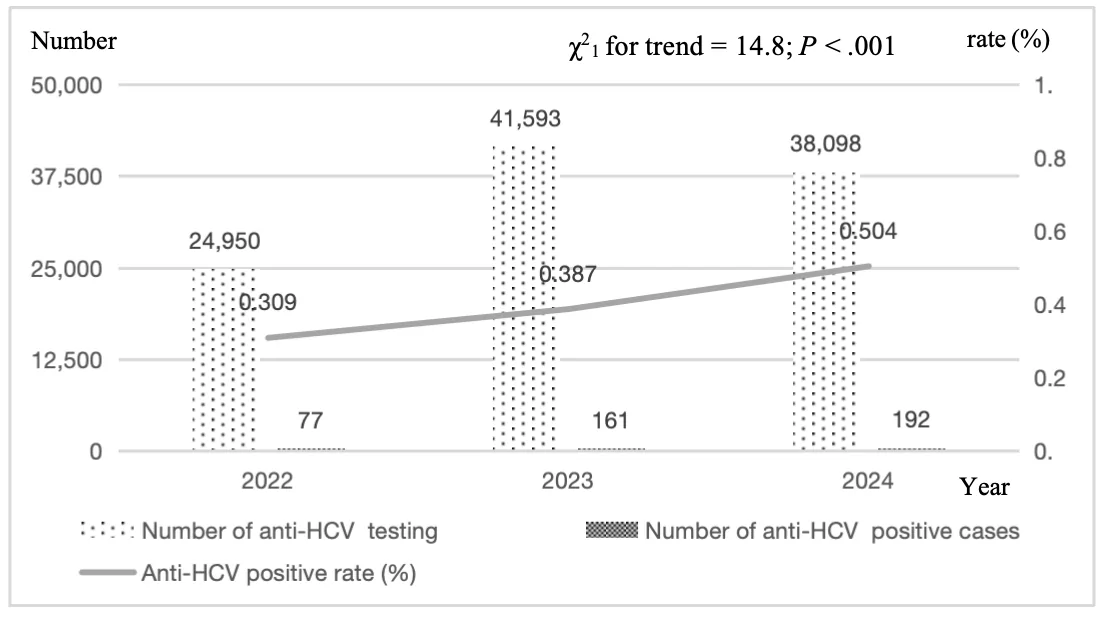
Trends in anti–hepatitis C virus (anti-HCV) test volume, anti-HCV–positive cases, and anti-HCV positivity rates among voluntary counseling and testing clients at Centers for Disease Control and Prevention–affiliated clinics in Zhejiang Province from 2022 to 2024. The statistical significance of the temporal trends was examined using the chi-square test for trend.

**Table 2:** Positivity rates for HIV, syphilis, and anti–hepatitis C virus (anti-HCV) coinfections among voluntary counseling and testing clients at Centers for Disease Control and Prevention–affiliated clinics from 2022 to 2024.

Year	HIV and anti-HCV				HIV and syphilis				HIV, syphilis, and anti-HCV		
	Testing number	Positive number	Positivity rate (95% CI)	Testing number		Positive number	Positivity rate (95% CI)	Testing number		Positive number	Positivity rate (95% CI)
2022	24,950	3	0.012 (0.003-0.035)	30,049		90	0.300 (0.240-0.360)	24,909		2	0.008 (0.001-0.029)
2023	41,593	1	0.002 (0.000-0.013)	44,951		112	0.249 (0.206-0.301)	41,563		0	0 (0)
2024	38,098	4	0.011 (0.003-0.027)	39,929		93	0.203 (0.189-0.295)	38,065		2	0.005 (0.001-0.019)

**Table 3:** Univariate and multivariable logistic regression analyses of the predictors associated with HIV, syphilis, and anti–hepatitis C virus (anti-HCV) positivity among voluntary counseling and testing clients at Centers for Disease Control and Prevention–affiliated clinics in 2024. Italicized values indicate 95% CIs that do not include 1.

Variables		HIV positivity				Syphilis positivity				Anti-HCV positivity		
		Positivity rate, n/N (%)	OR^a^ (95% CI)	aOR^b^ (95% CI)	Positivity rate, n/N (%)		OR (95% CI)	aOR (95% CI)	Positivity rate, n/N (%)		OR (95% CI)	aOR (95% CI)
**Counseling regions**												
	High-income	233/11,335 (2.1)	1	1	181/11,311 (1.6)		1	—^c^	12/9,892 (0.1)		1	1
	Middle-income	397/21,671 (1.8)	0.889 (0.755-1.047)	*1.388 (1.161-1.660)*	341/20,853 (1.6)		1.022 (0.852-1.226)	—	174/20,486 (0.8)		*7.053 (3.927-12.666)*	*3.568 (1.924-6.615)*
	Low-income	51/7,910 (0.6)	*0.309 (0.228-0.419)*	*0.411 (0.301-0.561)*	125/7,765 (1.6)		1.006 (0.800-1.266)	—	6/7,720 (0.1)		0.640 (0.240-1.707)	0.648 (0.237-1.771)
**Sex**												
	Male	601/32,228 (1.9)	1	1	490/31,459 (1.6)		1	—	136/29,895 (0.5)		1	1
	Female	80/8,688 (0.9)	*0.489 (0.387-0.618)*	*0.511 (0.385-0.676)*	157/8,470 (1.9)		1.194 (0.996-1.431)	—	56/8,203 (0.7)		1.504 (0.101-2.055)	*0.677 (0.476-0.963)*
**Age (years)**												
	16-34	290/19,611 (0.7)	1	1	234/19,086 (1.2)		1	1	12/18,199 (0.1)		1	1
	35-54	272/16,055 (1.7)	1.148 (0.972-1.357)	*1.329 (1.038-1.703)*	290/15,676 (1.8)		*1.518 (1.277-1.806)*	*1.541 (1.224-1.940)*	140/14,883 (0.9)		*14.392 (7.978-25.962)*	*10.360 (5.629* *-* *19.069)*
	≥55	119/5,250 (2.3)	*1.545 (1.246-1.917)*	*1.690 (1.235-2.312)*	123/5,167 (2.4)		*1.965 (1.576-2.449)*	*1.966 (1.473-2.624)*	40/5,016 (0.8)		*12.183 (6.387-23.241)*	*6.224 (3.154-12.281)*
**Marital status**												
	Unmarried	254/12,501 (2)	1	1	172/12,332 (1.4)		1	1	11/11,767 (0.1)		1	1
	Married	270/16,267 (1.7)	*0.807 (0.682-0.955)*	*0.565 (0.432-0.738)*	262/16,014 (1.6)		*1.219 (1.021-1.454)*	0.803 (0.638-1.011)	39/14,922 (0.3)		*5.109 (2.904-8.990)*	—
	Divorced or widowed	56/1,683 (3.3)	*1.562 (1.167-2.089)*	1.153 (0.803-1.657)	41/1,677 (2.4)		*1.615 (1.161-2.245)*	1.047 (0.584-1.473)	9/1,642 (0.6)		*5.943 (2.688-13.140)*	—
**Educational background**												
	Junior high school and under	307/12,910 (2.4)	1	1	268/12,697 (2.1)		1	1	48/11,973 (0.4)		1	—
	Senior high school	93/8,367 (1.1)	*0.480 (0.381-0.606)*	*0.455 (0.356-0.583)*	95/8,257 (1.2)		*0.624 (0.508-0.767)*	*0.665 (0.541-0.818)*	8/7,824 (0.1)		*0.448 (0.254-0.792)*	—
	College and above	165/8,000 (2.1)	0.909 (0.756-1.092)	*0.646 (0.515-0.811)*	93/7,910 (1.2)		*0.594 (0.477-0.739)*	*0.734 (0.584-0.922)*	3/7,387 (0.04)		*0.392 (0.228-0.672)*	—
**Consultation types**												
	Proactive	594/30,307 (2)	1	1	459/29,607 (1.6)		1	1	56/27,815 (0.2)		1	1
	Outreach	19/8,893 (0.2)	*0.107 (0.068-0.169)*	*0.096 (0.060-0.153)*	163/8,618 (1.9)		*1.224 (1.022-1.466)*	*1.241 (1.033-1.491)*	54/8,579 (90.6)		*3.140 (2.159-4.567)*	*2.837 (1.882-4.276)*
	Referral	68/1,716 (4)	*2.064 (1.598-2.666)*	*1.632 (1.249-2.131)*	25/1,704 (1.5)		0.946 (0.630-1.418)	0.952 (0.634-1.430)	82/1,704 (4.8)		*25.060 (17.776-35.329)*	*31.869 (20.557-49.406)*
**Consultation reasons**												
	Casual heterosexual behavior	126/11,972 (1.1)	1	1	189/11,823 (1.6)		1	—	33/11,570 (0.3)		1	1
	Homosexual behavior	385/17,601 (2.2)	*2.102 (1.717-2.574)*	*1.909 (1.514-2.408)*	264/17,024 (1.6)		0.970 (0.803-1.170)	—	5/15,713 (0)		*0.111 (0.043-0.285)*	*0.031 (0.011-0.082)*
	Having an HIV- positive intimate partner	89/2,077 (4.3)	*4.209 (3.195-5.544)*	*3.526 (2.546-4.882)*	45/2,034 (2.2)		1.393 (1.003-1.934)	—	7/1,980 (0.4)		1.240 (0.548-2.808)	0.826 (0.350-1.950)
	Blood exposure–related behaviors	3/241 (1.2)	1.183 (0.374-3.751)	1.615 (0.505-5.167)	1/235 (0.4)		0.263 (0.037-1.885)	—	0/184 (0)		N/A^d^	N/A
	Injection drug use	0/74 (0)	—	—	3/73 (4.1)		2.638 (0.823-8.453)	—	27/73 (37)		*205.204 (114.291-368.434)*	*30.495 (15.584-59.672)*
	Others	78/8,951 (0.9)	0.826 (0.622-1.098)	1.005 (0.748-1.351)	145/8,740 (1.7)		1.038 (0.835-1.292)	—	120/8,578 (1.4)		*4.960 (3.371-7.299)*	*2.591 (1.675-4.006)*
**HIV testing history**												
	No	253/21,601 (1.2)	1	1	311/21,162 (1.5)		1	1	69/20,679 (0.3)		1	1
	Yes	428/19,315 (2.2)	*1.912 (1.635-2.236)*	*1.455 (1.208-1.754)*	336/18,767 (1.8)		*1.222 (1.046-1.428)*	*1.294 (1.105-1.516)*	123/17,41 (90.7)		*2.124 (1.581-2.854)*	*2.943 (2.069-4.187)*

^a^OR: odds ratio.

^b^aOR: adjusted odds ratio.

^c^These variables were not included in the model.

^d^N/A: not available. These data were not available because the odds ratio could not be estimated due to 0 events or model convergence issues.

## Discussion

### Prevalence of HIV, Syphilis, and Anti-HCV and Coinfection

From 2022 to 2024, the HIV positivity rates among VCT clients at CDC-affiliated clinics in Zhejiang Province decreased from 2.462% to 1.664%. This positivity rate was higher than the national average of 0.82% for VCT clinics in 2022 [16]. A study conducted in Chiang Mai Province, Thailand, found that the HIV positivity rates among individuals attending fixed-site and mobile VCT services were 5.6% and 2.6%, respectively [17]. The findings of this study indicated an overall decreasing trend in the newly reported HIV positivity rates, which were consistent with the downward trend in the overall HIV epidemic in Zhejiang Province.

This study showed that the syphilis positivity rates among VCT clients at CDC-affiliated clinics increased from 1.378% in 2022 to 1.62% in 2024. The reported incidence of syphilis in Zhejiang Province was 35.23 per 100,000 in 2022, with a decreasing trend from 2016 to 2022 [18]. The positivity rates observed in this study were higher than those reported in other regions of China and in studies conducted in the United States (2016-2023) and the United Kingdom [19-21].

The results of this study demonstrated that the anti-HCV positivity rates among VCT clients at CDC-affiliated clinics increased from 0.309% in 2022 to 0.504% in 2024. This rate was lower than the 1.8% reported for VCT clinics in Beijing [22] and the 1.5% reported by Conway [23] among CDC clients at community-based clinics across multiple European countries in 2018. The positivity rates for anti-HCV in Zhejiang Province showed a decreasing trend, with rates of 5.13 per 100,000, 3.97 per 100,000, and 3.61 per 100,000 in 2022, 2023, and 2024, respectively.

A study conducted among female sex workers in South China reported that the positivity rates for dual syphilis–anti-HCV, HIV-syphilis, HIV–anti-HCV, and triple HIV-syphilis–anti-HCV coinfection were 30 per 100,000, 0%, 0%, and 0%, respectively [24]. The probability of coinfection varied across different populations. In contrast, the prevalence of dual HIV-HCV coinfection was 39.1% among people who inject drugs in Vietnam, 8.5% among prison inmates in India, and 4% among drug users in the United States [25-27]. In Mexico, the national positivity rates for syphilis, HIV, and their dual coinfection among men who have sex with men were 15.2%, 10.2%, and 5.7%, respectively [28]. In the present study, the positivity rates for triple HIV-syphilis–anti-HCV coinfection among VCT clients at CDC-affiliated clinics were extremely low, with wide 95% Clopper-Pearson CIs, indicating substantial statistical imprecision. Therefore, the results should be interpreted with caution.

### Possible Explanations for Prevalence and Coinfection

The variation in the overall testing volume during the study period was significantly influenced by the COVID-19 pandemic. The peak testing volume observed in 2023 might be attributable to COVID-19 prevention and control restrictions implemented throughout 2022, which potentially biased the observed temporal trends in positivity rates for HIV and syphilis [29].

The increase in the number of VCT clients who engaged in male-male sexual behaviors at CDC-affiliated clinics in this study could be attributed to the integration of counseling and testing services for men who have sex with men provided by CBOs into the VCT information system in 2024. Although the number of men who have sex with men undergoing testing increased, the HIV positivity rates did not rise, which may be attributable to the generally stable HIV prevalence among key populations.

The rising trend on the syphilis epidemic may be associated with the economic prosperity of eastern China. The impact of HIV postexposure prophylaxis (PEP) and preexposure prophylaxis (PrEP) on syphilis infection warrants consideration. Recently, the coverage of PEP and PrEP services has expanded continuously. Currently, all 11 prefecture-level cities in Zhejiang Province have established 24-hour PEP clinics, and advancements in internet technology have provided robust support for PrEP service delivery [30]. However, evidence from available data is insufficient and warrants further research.

The generally low positivity rates for anti-HCV and concurrent HIV and anti-HCV infection could be attributed to the relatively small proportion of people who inject drugs among VCT clients at CDC-affiliated clinics, along with the notable effectiveness of harm reduction programs, such as clean needle exchange programs and methadone maintenance therapy in Zhejiang Province [31]. Conclusions regarding coinfection patterns should be interpreted cautiously and restricted solely to VCT attendees.

### Correlates of HIV, Syphilis, and Anti-HCV Positivity Among VCT Clients at CDC-Affiliated Clinics

In the present study, an inverse association was observed between educational background and the risk of HIV, syphilis, and anti-HCV positivity. The association between education and infectious disease outcomes has been extensively examined in prior research, with a consistent protective effect reported across most studies [32]. Individuals with higher levels of education exhibited greater health consciousness, more access to health information, and higher use of health services and resources, which may contribute to a reduced risk of infection with the aforementioned infectious diseases.

Compared with individuals who actively sought services at CDC-affiliated VCT clinics, those who received referral-based testing had a higher risk of positivity for HIV–anti-HCV coinfection. In contrast, individuals receiving counseling and testing through outreach services had a lower risk of positivity for HIV-syphilis coinfection but a higher risk of anti-HCV positivity. Individuals who voluntarily attended CDC-affiliated VCT clinics for testing generally exhibited stronger risk awareness and self-protection capabilities, as well as higher levels of awareness regarding condom use [33]. On the other hand, referred individuals typically had lower risk awareness than those who actively sought services yet still engaged in high-risk behaviors, and this led to a higher infection rate. Outreach services are generally targeted toward hard-to-reach populations, such as community-based drug abstainers and those under supervision in correctional facilities, such as compulsory drug detoxification centers. This may explain the relatively high HCV infection rate observed in this subgroup.

Male homosexual behavior and having HIV-positive intimate partners were associated with HIV infection among VCT clients at CDC-affiliated clinics in our province. As our province had low HIV prevalence, sentinel surveillance data revealed that the HIV positivity rate among this population reached 4%, making it the high-risk group with the highest infection rate in the province [34]. Spousal notification and testing constituted a core component of HIV prevention and control efforts in the province. The HIV positivity rate was as high as 30% among spouses, which was attributed to delayed diagnosis of index patients [35].

Our study showed that individuals with an HIV testing history had a higher risk of HIV, syphilis, and HCV infection. A history of HIV testing was associated with risky behaviors and psychological traits; for example, people with high health awareness and those engaging in more high-risk behaviors are more likely to undergo HIV testing [36]. Moreover, a high frequency of high-risk behaviors was a significant risk factor for these 3 diseases, which may explain this result.

### Programmatic Implications for VCT and Integrated Disease Control

The findings of this study yield several implications for intervention practices. First, testing for HIV, syphilis, and HCV in VCT clinics offers important data for epidemic surveillance. However, some VCT attendees at CDC clinics were not tested for syphilis and HCV, and anti-HCV–positive individuals failed to receive nucleic acid testing, which hinders epidemic monitoring. Screening strategies for syphilis and anti-HCV, as well as HCV nucleic acid testing, need to be improved. Second, this study found that VCT clients recruited through outreach and referral had higher positivity rates for HIV, syphilis, and anti-HCV compared with walk-in clients. Therefore, VCT clinics should scale up outreach and referral testing services. CBOs and high-risk behavior intervention teams, which have access to hard-to-reach high-risk populations, should be engaged to promote VCT services. Third, although the findings of this study cannot draw definitive conclusions, we still suggest delivering more targeted and individualized interventions during counseling sessions for the older adults, male participants, unmarried individuals, people with low educational attainment, and populations identified via referral and outreach services, to reduce their risk of seroconversion.

### Limitations

There were many limitations in this study. First, follow-up nucleic acid testing results for individuals who screened positive for HCV were unavailable, which limited accurate estimation of the actual HCV infection rate among VCT clients at CDC-affiliated clinics. Second, from the perspective of factor exploration, this study was based on an analysis of existing data without a rigorous scientific theoretical hypothesis. This may have led to the omission of key confounding factors. Third, the participants were not recruited via random sampling, which limited the generalizability of the study findings to the general population or to all key populations. Fourth, testing services provided by VCT clinics are anonymous, so duplicate individual records may exist in theory. Although the large sample size can mitigate their impact on analytical results, we still recommend excluding duplicate cases in future studies. Finally, the MAR assumption could not be empirically verified, which was noted as a study limitation. Thus, future research with scientifically rigorous study designs is warranted, building on the key findings of the present study.

### Conclusions

From 2022 to 2024 in Zhejiang Province, the HIV and anti-HCV positivity rates among VCT clients at CDC-affiliated clinics declined, while the syphilis positivity rates increased. The coinfection positivity rates for these 3 infections remained at low levels. Individuals reporting homosexual behavior constituted a high-risk group for HIV yet exhibited a lower anti-HCV positivity rate. Among CDC-affiliated VCT clients, older age, lower educational attainment, and prior HIV testing history were associated with higher infection positivity rates, suggesting that these characteristics might help refine targeting of VCT and prevention efforts within this setting. It is suggested that VCT clinics enhance syphilis and HCV testing, expand outreach and referral services, and deliver targeted counseling and interventions for populations at high risk of infection.

## Abbreviations

aOR: adjusted odds ratio
ART: antiretroviral therapy
CBO: community-based organization
CD4: cluster of differentiation 4
CD8: cluster of differentiation 8
CDC: Centers for Disease Control and Prevention
ELISA: enzyme-linked immunosorbent assay
HCV: hepatitis C virus
IDU: injection drug use
LR: logistic regression
MAR: missing‑at‑random
OR: odds ratio
PEP: postexposure prophylaxis
PrEP: preexposure prophylaxis
RPR: rapid plasma regain
TPPA: Treponema pallidum particle agglutination assay
TRUST: toluidine red unheated serum test
VCT: voluntary counseling and testing

## Data Availability

The datasets used and/or analyzed during the current study are available from the corresponding author on reasonable request.
